# Enhanced Lateral Photovoltaic Effects in n-Si/SiO_2_/PEDOT:PSS Structures

**DOI:** 10.3390/polym14071429

**Published:** 2022-03-31

**Authors:** Jingying Zhang, Kang Meng, Gang Ni

**Affiliations:** 1 Department of Optical Science and Engineering, Fudan University, Shanghai 200433, China; 19210720002@fudan.edu.cn (J.Z.); 17210720144@fudan.edu.cn (K.M.); 2 Shanghai Engineering Research Center for Ultra-Precision Optical Manufacturing, Fudan University, Shanghai 200433, China

**Keywords:** lateral photovoltaic effect, hybrid solar cells, organic semiconductors, PEDOT:PSS

## Abstract

Organic/silicon hybrid structures have been extensively studied for the application of solar cells due to their high photoelectric conversion efficiency and simple fabrication process. However, studies of lateral photovoltaic effects (LPEs) in the devices are still scarce. Herein, the Si/SiO_2_/PEDOT:PSS devices were prepared by spin-coating, and showing the lateral photovoltage (LPV) sensitivity of 14.0 mV/mm at room temperature, which is higher than the control samples of Si/SiO_2_ (0.1 mV/mm) and Si/PEDOT:PSS (9.0 mV/mm) structures. With the decrease in temperature, the lateral photovoltage increases initially, and reaches a peak at around 210 K, then drops accordingly. The enhancement of LPE can be mainly ascribed to the formation of the p-n junction and the native oxide layer at the organic/inorganic interface.

## 1. Introduction

Recently, organic/silicon hybrid solar cells (HSCs) have attracted a lot of attention due to the combined advantages of the high carrier mobility of Si, the tailorable energy levels of conjugated polymers, and low-temperature processing techniques [1,2,3]. As a kind of typical p-type organic semiconductor material, poly(3,4-ethylenedioxythiophene):poly(styrene sulfonate) (PEDOT:PSS) has been widely used in HSCs. PEDOT:PSS can be spin-coated or ink-jet-printed on silicon substrate, forming a p-n stack structure for solar cell applications. At present, the Si/PEDOT:PSS HSCs have reached efficiencies beyond 17% [1].

Different from the conventional transverse photovoltaic effect used for solar cells, the lateral photovoltaic effect (LPE) refers to the phenomenon of generating a photovoltaic voltage between two electrodes on the same surface when a light beam illuminates the surface of the device [4,5]. In recent decades, due to the applications in position-sensitive detectors (PSD), LPE was widely investigated in a variety of silicon-based structures, such as metal-semiconductor junctions, metal-oxide-semiconductor junctions, and 2D materials-based heterojunctions [4,5,6,7,8,9]. Till now, the research on LPE has mainly been focused on inorganic devices, with only a few reports on organic LPE devices, such as Co-Alq_3_/Si, P3HT/Al, and ITO/PEDOT:PSS/MEH-PPV:PCBM/Al [10,11,12].

In comparison with intensive studies on Si/PEDOT:PSS solar cells, the LPEs of the hybrid heterojunction have received much less attention. Recently, M. Javadi et al. observed the LPE in Si/PEDOT:PSS structure and studied the influence of the thickness of the PEDOT:PSS layer and the wavelength on the device performance [13]. However, most of the above studies were only carried out at room temperature, and the temperature dependence of LPE is still unclear, which is important for the PSD applications at low temperatures. In this paper, we further investigated the LPE performance and the temperature dependence of LPE in n-Si/SiO_2_/PEDOT:PSS hybrid heterojunction, and discussed the possible mechanism.

## 2. Materials and Methods

The n-type Si(111) wafers (R: 1–10 Ω-cm, thickness: 3 mm) with native oxide layer (~2.3 nm) were cut into 5 mm × 10 mm slices, then were rinsed with deionized water and cleaned with acetone and ethanol ultrasonically. In the experiment, different from the high conducting PEDOT:PSS (PH1000, R < 0.001 Ω-cm) used in the previous report by M. Javadi [13], the lower conducting PEDOT:PSS solution (purchased in Clevios, AI 4083, R: 500–5000 Ω-cm) was spin-coated on the silicon substrates with native SiO_2_ layer at an angular speed of 1200 rpm, then thermally annealed at 150 °C for 15 min. Finally, the indium electrodes were placed on the surfaces of the devices, with a distance of about 6 mm.

**Figure 1:**
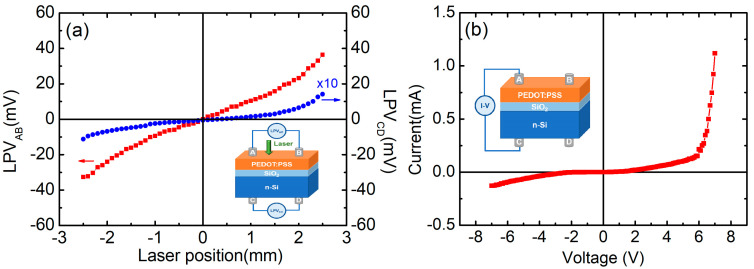
(**a**) The LPV dependence of laser position in the Si/SiO_2_/PEDOT:PSS; (**b**) I-V curve of the Si/SiO_2_/PEDOT:PSS. The schematic diagrams of the devices are shown in the insets.

The measurements were performed in a vacuumed optical cryostat (Cryo industries) at different temperatures (50–300 K). As is shown in the inset of Figure 1a, the samples were scanned with a semiconductor laser (10 mW, 532 nm) focusing on a roughly 200 μm diameter spot at the surface in the dark. Lateral photovoltage (LPV) curves and current-voltage (I-V) curve were measured by Keithley 2001 and 238 sourcemeter respectively. The thickness of the SiO_2_ layer on Si wafer was determined by an ellipsometer (J.A.Woollam, V-VASE).

## 3. Results and Discussion

Figure 1a shows the LPV as a function of the laser point position for the n-Si/SiO_2_/PEDOT:PSS structure at room temperature, where LPV_AB_ denotes the lateral photovoltage between the electrodes on the upper PEDOT:PSS organic surface, and LPV_CD_ denotes the lateral photovoltage on the Si side. A significant LPE can be observed in the sample. When the laser irradiation point moves from the electrode A to B, the LPV_AB_ (PEDOT:PSS side) and LPV_CD_ (Si side) exhibit approximately linear changes with a positive slope, with the LPV_AB_ and LPV_CD_ sensitivities of 14.0 mV/mm and 0.25 mV/mm, respectively.

The current–voltage characteristics of the n-Si/SiO_2_/PEDOT:PSS structure are shown in Figure 1b. Two electrodes were placed on the surfaces of PEDOT:PSS film and Si substrate separately. With the increasing voltage, the current increases nonlinearly and shows obvious rectifying characteristics. The rectifying behavior results from the p-n junction formed at the interface between the p-type PEDOT:PSS layer and the n-Si substrate, which has been proven in HSCs before [2,14].

In order to investigate the roles of the PEDOT:PSS layer and native oxide layer in the devices, we also studied the LPE of two control samples: one is Si/SiO_2_ (Si substrate with native oxide layer), and the other is Si/PEDOT:PSS (where the SiO_2_ layer was removed by HF acid).

**Figure 2:**
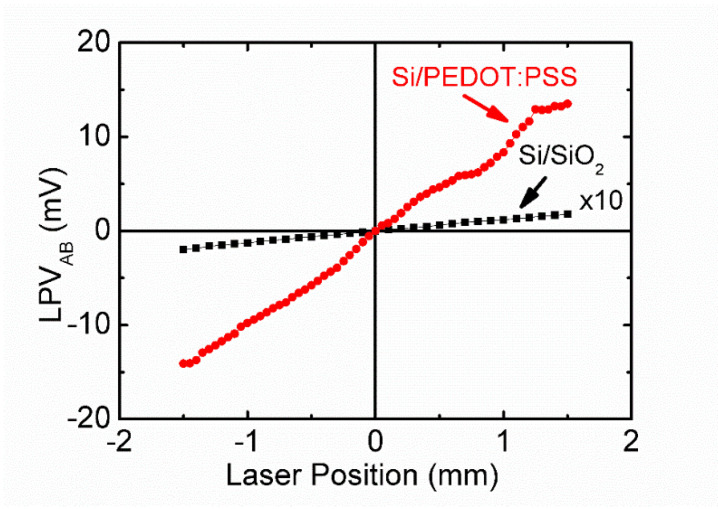
LPV_AB_ as a function of laser position in Si/SiO_2_ structure (black square dotted line) and Si/PEDOT:PSS structure (red circle dotted line).

The LPVs as a function of laser position in the Si/SiO_2_ and Si/PEDOT:PSS structures are presented in Figure 2, showing LPV sensitivities of about 0.1 mV/mm (Si/SiO_2_) and 9.0 mV/mm (Si/PEDOT:PSS) respectively, both of which are smaller than that in the Si/SiO_2_/PEDOT:PSS sample. The enhancement of LPE in Si/SiO_2_/PEDOT:PSS is mainly ascribed to the introduction of PEDOT:PSS layer, and the native oxide layer also plays a role in the LPE process. In addition, the linearity of the LPV curve is not very good for Si/PEDOT:PSS, which possibly results from the rough surface of Si substrate after the HF acid treatment.

**Figure 3:**
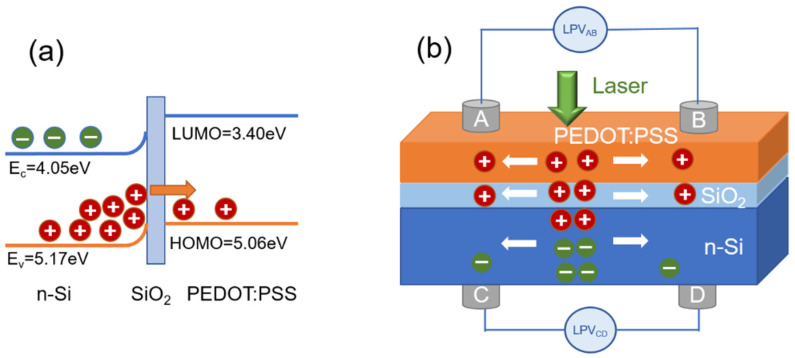
(**a**) The energy band diagram and (**b**) diagram of LPE mechanism in Si/SiO_2_/PEDOT:PSS.

Figure 3a shows the energy band diagram of the Si/SiO_2_/PEDOT:PSS structure. Due to the presence of PEDOT:PSS, a strong inversion layer was formed at the organic/inorganic interface [2]. Besides, owing to the SiO_2_-Si bonding, a positive surface dipole was formed at the interface, thus bending up the Si energy band and aligning to the PEDOT:PSS energy band, further improving carrier separation at the interface [15].

As seen in Figure 3b, when a laser beam is irradiated on the sample, photo-generated electron-hole pairs are generated mainly in the Si substrate because of the transparent property of the PEDOT:PSS layer. Due to the built-in field between the p-type PEDOT:PSS layer and the n-Si substrate, the electron-hole pairs are separated, the photo-generated holes are transferred to the upper organic layer, and the electrons remain in the Si substrate. Furthermore, a carrier concentration gradient between the illumination and non-illumination zone is formed, and thus the carriers diffuse laterally, finally leading to the position-dependent lateral photovoltage [4,9].

Based on the carrier diffusion model, LPV can be described as [9,16]:

(1) $$LPV=KN_{0}\left[\exp\left(-\frac{\left|\frac{L}{2}-x\right|}{\lambda }\right)-\exp\left(-\frac{\left|\frac{L}{2}+x\right|}{\lambda }\right)\right]\approx \frac{2KN_{0}}{\lambda }\exp\left(-\frac{L}{2\lambda }\right)x$$

where *K* is the proportionality coefficient, *L* is the distance between two electrodes, *N*_0_ is the number of separated electron-hole pairs per second, and *λ* is the carrier diffusion length.

According to Equation (1), the enhanced LPV in Si/SiO_2_/PEDOT:PSS results from an efficient carrier separating process, low recombination rate, and appropriate carrier diffusion length. Firstly, the thin SiO_2_ layer can passivate the Si surface dangling bonds, thus promoting carrier separation and suppressing the carrier recombination at the Si interface [2,8,17]. Secondly, compared with the Si/SiO_2_, the stronger built-in field can also improve the hole injection into the organic layer for the Si/SiO_2_/PEDOT:PSS sample. Thirdly, the relatively large resistance of the organic layer will decrease the hole diffusion length and increase the electric potential gradient, then enhance the LPE. In addition, the small LPV_CD_ of the Si side in Si/SiO_2_/PEDOT:PSS possibly results from the relatively low resistance of the Si wafer.

**Figure 4:**
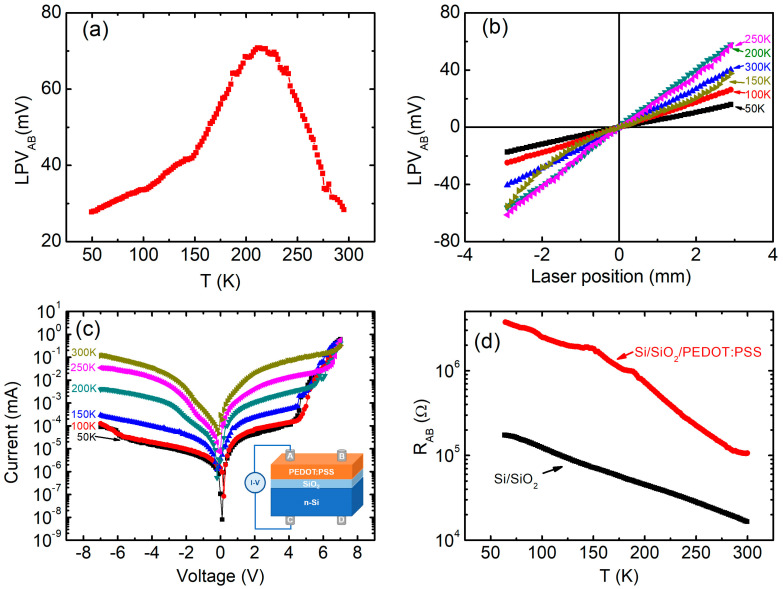
(**a**) Temperature dependence of LPV_AB_, (**b**) LPV_AB_ curves and (**c**) I-V curves for Si/SiO_2_/PEDOT:PSS at different temperatures. (**d**) Temperature dependence of resistances (R_AB_) of Si/SiO_2_/PEDOT:PSS and Si/SiO_2_.

The influence of temperature on the LPE of Si/SiO_2_/PEDOT:PSS was investigated, as shown in Figure 4a. The LPV was measured on the fixed position of 2 mm from the centre of the electrodes with the temperature sweeping from 300 K to 50 K. With the decrease in temperature, the LPV increases gradually from 28.1 mV(300 K) to 70.8 mV (210 K), then finally decreases to 27.9 mV (50 K). Figure 4b presents LPV curves of the sample at different temperatures, showing the typical LPE behaviours with good linearities.

As can be seen from Equation (1), LPV is mainly determined by the number of separated electron-hole pairs *N*_0_ and the carrier diffusion length *λ*, both of which are temperature-dependent. In order to understand the above temperature dependence of LPE, the *I*-*V* curves of the sample were measured at different temperatures, as shown in Figure 4c, where the vertical axis is on a logarithmic scale. As the temperature decreases, the rectifying behaviour obviously increases, indicating a stronger built-in field and more separated electron-hole pairs, thus leading to the enhancement of LPE at low temperatures.

Figure 4d presents the temperature dependences of the resistances (R_AB_) for Si/SiO_2_/PEDOT:PSS and Si/SiO_2_ samples, where two electrodes (A and B) are on the top surface of the samples. With the decrease in temperature, both of the resistances for the two samples increase at a rough exponential rate, showing the typical conducting behaviour of semiconductors. As we know, the diffusion length λ of carriers can be written as [16]:

(2) $$\lambda =\sqrt{\frac{3k_{B}T\tau }{8\pi q^{2}\rho }{\left(\frac{2mE_{F}}{\hbar^{2}}\right)}^{-\frac{3}{2}}}$$

According to Equations (1) and (2), we can obtain LPV sensitivity, which is proportional to *λ*^−1^*exp*(−*L*/2*λ*) or *ρ*^1/2^*exp*(−*Lρ*^1/2^/2). Since the diffusion length *λ* >> *L* at room temperature [16], the exponential term (*exp*(−*L*/2*λ*)) can be omitted near 300 K. With the initial drop in temperature (from 300 K to 210 K), the diffusion length λ decreases due to the increasing resistivity. Consequently, the separated holes diffuse slowly in the upper layer at low temperature, resulting in a high potential distribution in the plane of the film, showing increasing LPV from 300 K to 210 K. However, at a lower temperature (below 210 K), due to the rapid increase of resistivity, the exponential term (*exp*(−*L*/2*λ*)) plays an increasingly important role, and LPV begins to decrease with the further drop of temperature. The experimental results will help us to reveal the mechanism of LPE in Si/organic hybrid devices, and explore the potential applications of LPE-based temperature sensors.

## 4. Conclusions

In conclusion, the LPE of Si/SiO_2_/PEDOT:PSS structure has been studied at different temperatures. The LPV sensitivity of the device is 14.0 mV/mm at room temperature. The built-in field and the native oxide layer at the interface play important roles in the performance of LPE. With the decrease of temperatures from 300 K, LPV increases first, then decreases to below 210 K accordingly, which is mainly related to the temperature dependence of resistance for the organic layer. Due to its simple fabrication processes and high position sensitivity, the hybrid device can be a promising candidate for PSDs.
